# Intracycle Velocity Variation During a Single-Sculling 2000 m Rowing Competition

**DOI:** 10.3390/s25154696

**Published:** 2025-07-30

**Authors:** Joana Leão, Ricardo Cardoso, Jose Arturo Abraldes, Susana Soares, Beatriz B. Gomes, Ricardo J. Fernandes

**Affiliations:** 1 Centre of Research, Education, Innovation and Intervention in Sport, Faculty of Sport, University of Porto, 4200-450 Porto, Portugal; up202005623@up.pt (J.L.); susana@fade.up.pt (S.S.); ricfer@fade.up.pt (R.J.F.); 2 Porto Biomechanics Laboratory, Faculty of Sport, University of Porto, 4200-450 Porto, Portugal; 3 Research Group Movement Sciences and Sport (MS&SPORT), Department of Physical Activity and Sport, Faculty of Sport Sciences, University of Murcia, 30100 Murcia, Spain; abraldes@um.es; 4 CIPER (Interdisciplinary Center for the Study of Human Performance), Faculty of Sport Sciences and Physical Education (FCDEFUC), University of Coimbra, 3040-248 Coimbra, Portugal; beatrizgomes@fcdef.uc.pt

**Keywords:** rowing, biomechanics, intracycle velocity variation

## Abstract

Rowing is a cyclic sport that consists of repetitive biomechanical actions, with performance being influenced by the balance between propulsive and resistive forces. The current study aimed to assess the relationships between intracycle velocity variation (IVV) and key biomechanical and performance variables in male and female single scullers. Twenty-three experienced rowers (10 females) completed a 2000 m rowing competition, during which boat position and velocity were measured using a 15 Hz GPS, while cycle rate was derived from the integrated triaxial accelerometer sampling at 100 Hz. From these data, it was possible to calculate distance per cycle, IVV, the coefficient of velocity variation (CVV), and technical index values. Males presented higher mean, maximum and minimum velocity, distance per cycle, CVV, and technical index values than females (15.40 ± 0.81 vs. 13.36 ± 0.88 km/h, *d* = 0.84; 21.39 ± 1.68 vs. 18.77 ± 1.52 km/h, *d* = 1.61; 11.15 ± 1.81 vs. 9.03 ± 0.85 km/h, *d* = 1.45; 7.68 ± 0.32 vs. 6.89 ± 0.97 m, *d* = 0.69; 14.13 ± 2.02 vs. 11.64 ± 1.93%, *d* = 2.06; and 34.25 ± 4.82 vs. 26.30 ± 4.23 (m^2^/s·cycle), *d* = 4.56, respectively). An association between mean velocity and intracycle IVV, CVV, and cycle rate (*r* = 0.68, 0.74 and 0.65, respectively) was observed in males but not in female single scullers (which may be attributed to anthropometric specificities). In female single scullers, mean velocity was related with distance per cycle and was associated with technical index in both males and females (*r* = 0.76 and 0.66, respectively). Despite these differences, male and female single scullers adopted similar pacing strategies and CVV remained constant throughout the 2000 m race (indicating that this variable might not be affected by fatigue). Differences were also observed in the velocity–time profile, with men reaching peak velocity first and having a faster propulsive phase. Data provided new information on how IVV and CVV relate to commonly used biomechanical variables in rowing. Technical index (*r* = 0.87): distance per cycle was associated with technical index in both males and females (*r* = 0.76 and 0.66, respectively). Future studies should include other boat classes and other performance variables such as the power output and arc length.

## 1. Introduction

Rowing is a cyclic and symmetrical sport characterized by repetitive movement cycles involving coordinated movements of both the upper and lower limbs, with the oars functioning as levers that transmit force to the water and generate propulsion to accelerate the boat [1,2,3]. Each rowing cycle can be divided into two distinct phases: the propulsive phase, which starts when the blade enters the water (catch) and ends when it exits (finish), and the recovery phase, which spans from blade exit to its next water entry, during which no propulsion is produced and the rower repositions the body for the subsequent cycle [2,4]. The only external propulsive force applied to the system is produced through blade–water interaction during the drive phase, while hydrodynamic drag (comprising both viscous and wave resistance) acts continuously throughout the rowing cycle [5,6,7].

Effective propulsion results from the ability to maximize force application during the drive while minimizing resistive losses during recovery, making rowing efficiency highly dependent on the optimization of cycle mechanics and temporal coordination. Consequently, rowing performance is multifactorial, influenced by the rower’s physiological profile (e.g., aerobic and anaerobic capacities and muscular strength), anthropometric characteristics (e.g., limb length and body mass), and biomechanical execution (e.g., cycle length, force curve profile, timing, and blade trajectory) [8,9,10]. These interrelated components underscore the importance of an integrated approach to training and technique development in competitive rowing.

Advances in biomechanical instrumentation have improved the accessibility, reliability, and validity of data collection, enabling precise on-water technical assessments [11,12]. High-frequency sensors and GPS-integrated systems allow real-time analysis of kinematics, kinetics, and boat dynamics, providing coaches and researchers data that allows performance enhancement [7,12]. These tools have enabled a more detailed examination of the rowing cycle, including velocity variations within each cycle. In this context, there has been growing interest in transferring biomechanical performance indicators from other cyclic sports to rowing, particularly those developed and validated in swimming and canoeing.

The quantitative analysis of technique in cyclic sports (such as swimming and canoeing) has gained increasing interest, leading to a deeper understanding of velocity behavior during competition and training [13,14,15]. The concept of the technical index, originally introduced in swimming, is defined as the product of swimming velocity and distance per cycle. A swimmer with a high technical index covers more distance per cycle at higher velocity, suggesting better propulsion. This index offers a biomechanical indicator of global technical proficiency that is applicable across cyclic sports where movement efficiency is critical to performance [12,16,17]. In addition, research on intracycle velocity variation (IVV) in swimming has demonstrated an evident association between IVV and velocity, indicating that lower values of this variable corresponds to greater technical proficiency [18,19].

IVV is associated with the interaction between the cycle propulsive and recovery phases, and its minimization is generally regarded as beneficial for enhancing efficiency, ultimately contributing to higher average boat velocity and improved race performance [11]. In addition, IVV in rowing has been related to cycle rate, since its increase often resulting in elevated inertial forces and greater accelerations of both the rowers body mass and the boat, thereby influencing the magnitude of velocity fluctuations throughout the rowing cycle [20]. A deeper understanding of the interactions among these variables provides valuable insights into performance and energy efficiency in rowing, but research on this topic remains limited, since most studies have not been conducted in real competitive environments or over race distances typical of elite performance. Furthermore, research on the relationships between IVV and other biomechanical variables critical to rowing performance, particularly over a 2000 m race, remains limited. The aim of the current study was to assess the IVV of single scullers during a 2000 m competition and examine its relationships with other relevant biomechanical variables. We hypothesized that IVV would be strongly associated with velocity and cycle rate in both male and female rowers, with potentially distinct relationships between sexes.

## 2. Materials and Methods

Twenty-three rowers (10 females), all with significant experience in national and international regattas and regular training volumes of 10–20 h per week, volunteered for to participate in this study. Twenty-three races were recorded during three national regattas held at a high-performance rowing venue. Boat position, velocity, and acceleration were obtained using a 15 Hz GPS unit with 12 channel receivers and a tri-axial accelerometer sampling at 100 Hz (GPSPORT, Canberra, Australia) both integrated within the same device mounted on the boat bow deck [12,21]. Environmental conditions at the start and finish of each trial were recorded using a weather station (NK 5400, Kestrel, Boothwyn, PA, USA) positioned at water level and measured at the finish line. All participants provided informed consent prior to commencement of this study and signed an informed consent in accordance with the local research ethics committee and the Declaration of Helsinki.

Data were downloaded into a computer and analyzed using a validated custom-made MATLAB (version 2023a; Mathworks Inc., Natick, MA, USA) algorithm [21]. The race time (mean duration), mean velocity (ratio of distance to completion time), and cycle rate (mean number of cycles per min) were obtained from the collected data. From these primary variables, additional metrics were derived: distance per cycle (ratio of average velocity to the corresponding cycle rate), IVV (difference between maximum and minimum velocity within each cycle), coefficient of velocity variation (CVV; ratio of standard-deviation (SD) to mean velocity), and technical index (product of distance per cycle and velocity) [17]. The mean velocity–time profile was then calculated as the average of all complete cycles (including both propulsion and recovery phases) of each race.

All variables were calculated as mean and SD values for the complete race and per 100 m splits. Data normality and homogeneity were assessed using the Kolmogorov–Smirnov and Levene tests. All statistical procedures were performed using SPSS 27 (Chicago, IL, USA) and a 5% significance level was set. An independent sample *t*-test was conducted to identify sex-related differences with Cohen’s d effect sizes categorized as trivial (<0.2), small (0.2–0.6), moderate (0.6–1.2), large (1.2–2.0), and very large (>2.0) [22]. Pearson relation coefficients were used to evaluate linear relations between variables and interpreted as large (0.5–1.0), medium (0.3–0.5), and small (0.1–0.3). A repeated-measures ANOVA with Bonferroni post hoc tests was used to compare distance per cycle, velocity, IVV, and cycle rate across all 100 m splits for each sex. To compare the mean velocity profile between male and female rowers, Statistical Parametric Mapping (SPM) with independent two-sample *t*-test was conducted in MATLAB using SPm1d toolbox [23], with all statistical analyses being conducted in SPSS 27 (Chicago, IL, USA).

## 3. Results

During the 2000 m competition, male rowers demonstrated higher mean, maximum, and minimum velocity, distance per cycle, technical index, and CVV values as presented in Table 1. The Pearson correlation coefficients for each variable, for both male and female rowers, are displayed in Table 2. A large association was observed between the mean, maximum, minimum velocity, cycle rate, and IVV for male rowers. In addition, maximum velocity showed a large relation with both CVV and IVV, the latter being largely related with cycle rate in male rowers. For female single scullers, mean velocity was largely related with maximum and minimum velocity and distance per cycle. For both groups, cycle rate exhibited an inverse relation with distance per cycle and technical index.

**Figure 1:**
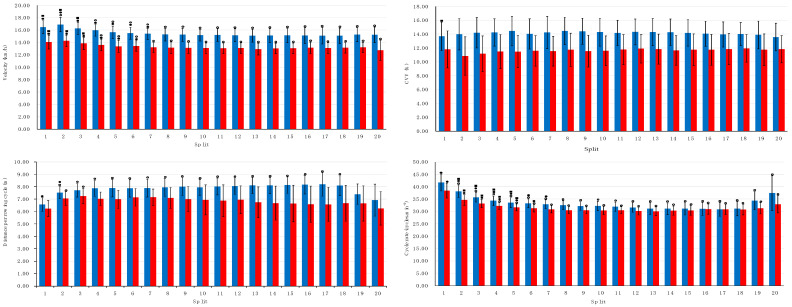
Mean ± SD values of velocity and CVV (**upper left** and **right panels**), as well as distance per cycle and cycle rate (**lower left** and **right panels**) for the different segments of the competition for male and female single-scullers (blue and red, respectively), ⚬, ● and ■ corresponds to differences between the indicated split and the 1st, the 10th and the 20th splits (respectively) (*p* < 0.05).

**Figure 2:**
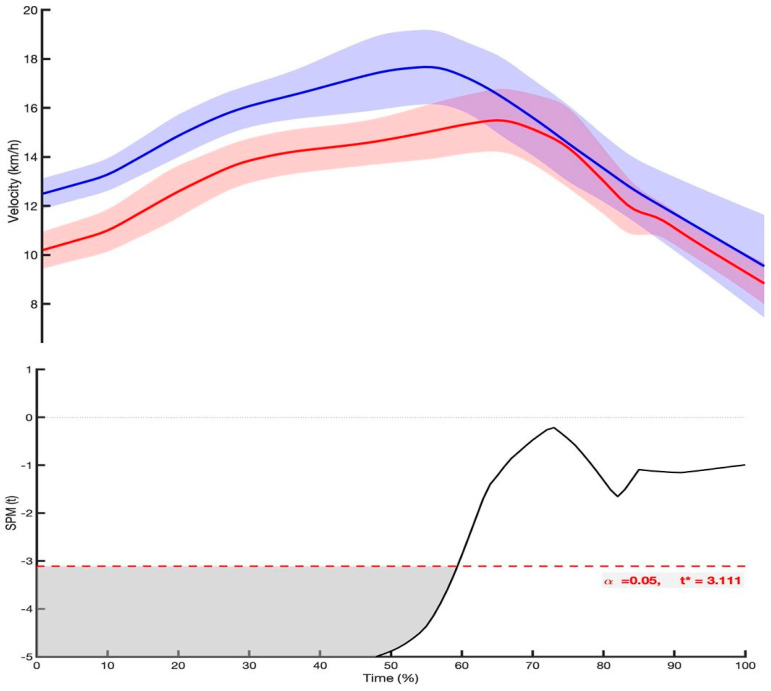
Mean ± SD of the mean velocity–time profile per rowing cycle of male and female single-scullers (**upper panel**, blue and red lines, respectively) and the independent two-sample *t*-test profile curve with the limits of significance (red dashed lines) displaying the differences between male and female rowers (**lower panel**). * ≤ 0.05.

Data on mean velocity, cycle rate, distance per cycle and CVV values, regarding the 100 m split segments of the race are displayed in Figure 1. Mean velocity values presented a slight decrease over the course of the competition, as differences were present between the 1st and the 10th and 20th splits for both groups. Both sexes presented stable values of CVV across splits, with males presenting higher values than females. The distance per cycle values were constant for female but not for male rowers, with differences being found between the 1st and the following 17 splits. The cycle rate values also presented changes over the competition in both groups with differences being found between 1st and the 10th and 20th splits. The rowing cycle velocity–time and the *t*-test profile are presented in Figure 2 (upper and lower panel, respectively). Male rowers presented higher velocity than females, particularly during the first half of the cycle (~0–60%), in addition higher SD in the final phase of the cycle (from ~80–100%) was also observed.

## 4. Discussion

Analyzing the velocity profile of a rower provides valuable insights and can highlight distinct technical strategies to optimize performance [11,24]. In rowing, increased water resistance is associated with higher IVV values, whereas maintaining a more constant velocity may enhance efficiency [12,20]. In the current study, differences in mean and maximum velocities were observed between male and female single scullers likely reflecting the former greater force production capacity [25,26]. Previous research indicates that male rowers tend to apply greater force over a shorter period, leading to higher peak velocity followed by a greater decline, whereas female rowers distribute their force more evenly, resulting in smaller velocity variations and a longer propulsive phase [24,25,27,28]. Moreover, male single scullers demonstrated a higher technical index, indicating that more distance is covered per cycle at a higher velocity. This reflects a more efficient application of force, which is particularly important for maintaining performance throughout a 2000 m race [16,17,18,29].

Our data supported the initial hypothesis that IVV would have a large relation with mean velocity and cycle rate, even if this association was only observed on male rowers. As previously reported, male rowers typically generate higher absolute power outputs and greater peak forces, resulting in higher mean velocity but also greater variations in velocity during each cycle, possibly explaining the large relation between IVV and mean velocity for men but not for women [30,31]. In addition, male rowers typically have higher body mass, which could lead to different momentum and inertia effects, amplifying the relation between IVV and cycle rate [32,33].

We also observed that cycle rate had a large and a direct relation with mean velocity for men and no association for women, with previous studies indicating that male rowers rely more on a higher cycle rate to generate velocity likely due to their ability to sustain higher power output over repeated cycles [34]. These findings are similar to those reported in canoeing, revealing that cycle rate has a greater impact on male performance compared to women [13,35]. This suggests that some technical metrics traditionally used in rowing may not have uniform effects across sexes, potentially affecting efficiency differently in female rowers. In fact, previous research has already pointed out the necessity of adapting the cycle rate according to the power output, aiming to preserve technical efficiency and benefit overall performance [12,27,29,36].

Regarding the different race segments, the observed biomechanical variables presented distinctive behaviors. In the first two splits of the competition, males covered less distance per cycle compared to the remaining of the race, while women presented constant values throughout the race. Recent studies highlighted sex-based differences in pacing and effort distribution during 2000 m rowing races, with males generally exhibiting higher velocity and cycle rate in beginning of the race, while females tend to adopt more even pacing strategies [26,37]. However, in our data, both male and female single scullers displayed similar velocity profiles across race segments, suggesting comparable pacing strategies despite other sex-based biomechanical differences.

Studies in canoeing have observed consistent distance per cycle values during a 200 m maximal effort, whereas in rowing previous research indicates that the 2000 m distance and lower cycle rates may influence how athletes manage their technique [12,14,28]. Cycle rate was higher at the beginning of the race, followed by a period of stabilization in both groups, with this early increase being also verified among rowers in previous studies (since a higher cycle rate is typically used to accelerate the boat and achieve greater velocity [37]). The constant CVV values through the race for both groups might indicate that this variable was not affected by the changes on other biomechanical variables, neither by the presence of fatigue.

The observed mean cycle velocity–time profile suggests a similar curve for both male and female rowers, indicating comparable approaches to velocity regulation throughout the cycle. The beginning of the curve represents the instant when the boat starts to experience positive acceleration, immediately after the water entry and concludes with the subsequent cycle when the boat resumes accelerating. Male rowers exhibited higher velocities than their female counterparts throughout the rowing cycle, particularly during the first half of the cycle (~0–60%), representing mainly the cycle propulsive phase. The location of the peak velocity indicates the time taken to reach its maximum value with male single scullers reaching peak velocity in less time, likely due to their greater power output compared to females (as noted on previous studies [29,38]).

No differences were observed between groups during the final phase of the velocity–time profile, corresponding to the recovery phase and the entry of the blades into the water. However, male rowers presented higher SD, possibly due to the higher mass moving towards the catch phase. Also, there might be small differences in how each male rower controls this mass, particularly during the deceleration and repositioning of the body. These subtle variations in technical execution (such as differences in the timing of trunk and arm movement during recovery) can result in more fluctuation in velocity, particularly at higher cycle rates [28,39]. Even if the current study presents original data and relevant outcomes, it presents some limitations. The inclusion of data on other biomechanical variables (such as rowing arc length and power) could provide a more comprehensive understanding of rowing mechanics and performance differences between genders. We acknowledge that the absence of direct anthropometric data (e.g., body mass, height, or limb length) in our study represents a limitation, as these factors are known to influence dynamics and overall mechanical efficiency.

## 5. Conclusions

The current study provides new information on how IVV relates to commonly used biomechanical variables in rowing. Male rowers exhibited greater CVV due to their ability to generate higher peak forces in a short duration effort, while female rowers demonstrate a more even force distribution leading to steadier velocity through the rowing cycle. CVV remained stable across all race segments despite variations in other biomechanical variables, indicating that it may not be affected by fatigue. The mean cycle velocity–time profile was similar in both groups, with male rowers showing higher velocity through the propulsive phase of the cycle (also reaching peak velocity earlier) compared to females. These findings suggest that coaches should consider adjusting training strategies to adopt a more efficient race strategy. Since studies on IVV and CVV during competition are scarce, the current study provides a starting point for future research that should include other boat classes in a competitive setting, using specialized boat instrumentation to measure other rowing-specific variables like force and arc length.

## Figures and Tables

**Table 1:** Mean ± SD of the selected biomechanical variables during the single sculling 2000 m competition.

Variables	Males	Females	*p*	Effect Size (Cohen’s d)
Mean velocity (km/h)	15.40 ± 0.81	13.36 ± 0.88	<0.001	0.84
Maximum velocity (km/h)	21.39 ± 1.68	18.77 ± 1.52	<0.001	1.61
Minimum velocity (km/h)	11.15 ± 1.81	9.03 ± 0.85	0.002	1.45
Cycle rate (cycles.min^−1^)	33.65 ± 1.63	32.40 ± 4.09	0.343	0.30
Distance per cycle (m)	7.68 ± 0.32	6.89 ± 0.97	0.015	0.69
IVV (km/h)	5.78 ± 1.05	5.50 ± 1.11	0.521	1.08
CVV (%)	14.13 ± 2.02	11.64 ± 1.93	0.010	2.06
Technical Index (m^2^/s·cycle)	34.25 ± 4.82	26.30 ± 4.23	<0.001	4.56

**Table 2:** Pearson linear relation coefficients for each variable for male (M) and female (F) single-scullers.

Variables	Mean Velocity	Maximum Velocity	Minimum Velocity	Cycle Rate	Distance Per Cycle	Intracycle Velocity Variation (IVV)	Coefficient of Velocity Variation (CVV)
	M/F	M/F	M/F	M/F	M/F	M/F	M/F
Mean velocity	-						
Maximum velocity	0.59 */0.55 *						
Minimum velocity	0.64 */0.74 *	−0.03/0.66 *					
Cycle rate	0.65 */0.37	0.28/0.21	0.49/0.20				
Distance per cycle	0.40/0.42	0.4/0.024	0.17/0.21	−0.41/−0.56 *			
Intracycle velocity variation (IVV)	0.68 */0.17	0.61 */0.72 *	0.27/0.28	0.63 */0.09	0.13/−0.01		
Coefficient of velocity variation (CVV)	0.74 */0.34	0.70 */0.32	0.40/−0.11	0.68 */0.19	0.15/−0.11	0.59 */0.77 *	
Technical index	0.35/0.87 *	0.62 */0.58 *	0.03/0.76 *	−0.22/−0.11	0.76 */0.66 *	0.09/0.19	0.09/−0.18

* *p* ≤ 0.05.

## Data Availability

Data is contained within the article.
